# Editorial: Immunoregulation at mucosal surfaces

**DOI:** 10.3389/fimmu.2022.983201

**Published:** 2022-08-10

**Authors:** Javier Leceta, Rosa Del Campo, Stefan Jordan, Christoph S. N. Klose

**Affiliations:** ^1^ Department of Cell Biology, Faculty of Biology, Complutense University of Madrid, Madrid, Spain; ^2^ Servicio de Microbiologia, Hospital Universitario Ramon y Cajal and Instituto Ramon y Cajal de Investigación Sanitaria, Madrid, Spain; ^3^ Department of Microbiology, Infectious Diseases and Immunology, Charité – Universitätsmedizin Berlin, Corporate Member of Freie Universität Berlin and Humboldt-Universität zu Berlin, Berlin, Germany

**Keywords:** mucosal, innate immune system, adaptive immune system, immunoregulation, microbiota

Mucous membranes are the largest contact site between the external environment and the internal milieu. They protect the body, allow the exchange of nutrients or respiratory gases and are the barrier that ensures the integrity of the internal environment. These barriers are also subject to aggression from infectious agents, toxins, and physical trauma. They are also sites colonized by an abundant microbiota. Mucous membranes are defensive barriers in which three layers can be defined. The epithelium ensures selective integrity and impermeability by the presence of adhesive junctions between their cells. On the outside of this layer there is a layer of mucus and other secretions that serves as a barrier to a commensal symbiont microbiota, which provides micronutrients vitamins and regulatory molecules. The microbiota lives in symbiosis with the organism but occasionally this microenvironment can be colonized by pathogenic species. To deal with these aggressions, a third layer of this defense barrier is the immunological barrier associated with the mucous membranes. Mucous membranes house the largest population of immune cells in the entire body. The immune system associated with the mucous membranes recognizes pathogens and toxins from outside the barrier but must react to pathogens and tolerate the commensal microbiota. Their different components also receive signals from the other components of the mucosal barrier. All the components of the barrier weave a network of mutual interactions that ensure homeostasis, not only of the mucous membranes but also of the whole organism. Imbalances in the response of the different components of these circuits are the basis of a large number of diseases, not only of the systems in which they are located, but also on the whole-body systems. This Research Topic addresses different aspects of their components and their mutual interrelationships.

## The immunological barrier

Around 70% of the immune cells of the whole organism are associated with the mucous membranes. Among these cells are elements of both the innate immune system and the adaptive immune system.

### The innate immune system at mucosal surfaces

The different cell types of innate immunity include phagocytic cells (macrophages), eosinophils, dendritic cells (DCs), mast cells, and innate lymphoid cells (ILCs) that recognize environmental signals and microbes. Innate immune cells are the first to be activated by receptors that recognize molecular patterns (PAM) present in different microbes, but also by micronutrients provided by the commensal microbiota and ligands for aryl hydrocarbon receptor (AhR), retinoic acid, vitamin A and short chain fatty acids (SCFA), which interact with the different classes of ILCs in the intestine and lung and are reviewed by Shi et al. Once activated, they secrete cytokines and interact with cells of the adaptive immune system, directing the differentiation of distinct classes of T lymphocytes, whose migration to the different lymphoid organs influences the type of immune response, both locally and systemically.

Mucosal barriers are particularly enriched in unconventional lymphocytes, defined as lymphocytes that lack organized antigen receptors or express antigen receptors with a limited repertoire. Among these cells of the innate immune system are ILCs, mucosal-associated invariant T cells (MAIT), and Tγδ cells. The review by Cox et al. highlights the critical roles of unconventional T cells in regulating the barrier function, particularly in their repair, in both gut and lungs. The cells of the innate immune system are activated, not only by PAMs but also by alarmins, released by damaged epithelial cells. In a model of intestinal inflammation induced by dextran sulfate sodium (DSS), Phuong et al. describe that overexpression of IL-33 in this model does not promote inflammation but increases the expression of genes of Th2 responses, highlighting the role of exogenous application of IL-33 for intestinal inflammation promoting the activation of ILC2. The review by Valle-Noguera et al. summarizes the importance of ILC3 cells in the defense against pathogens (fungi, bacteria, and virus) in different mucous membranes (oral mucosa, intestine, and respiratory mucosa). ILC3s are activated by gut hypoxia conditions and by regulatory molecules from the microbiota and diet. The cytokines IL-17 and IL-22 produced by them, together with Tγδ cells, are effective against several pathogens in different locations. In this way, ILCs work together with the cells of the adaptive immune response to organize the most appropriate immune response to each pathogen and each location of the organism. The role of unconventional T cells is also the subject of the article by Qiu et al., where these authors highlight the role of MAITs in chronic obstructive pulmonary disease (COPD). MAITs migrate to inflamed tissues and modulate the immune response. In COPD these cells are very abundant in the lung and produce IL-17, skewing the immune response towards a Th17 profile, but not Th1. Two other articles highlight the importance of Tγδ cells in innate mucosal responses. Yang et al. show the participation of a subtype of these cells, Tγδ17, in acute lung inflammation due to particulate matter, characterized by neutrophil infiltration. The IL-17 produced by these cells, predominant in the airways, attract neutrophils and their presence depends on the commensal microbiota of the intestine, since it is not produced in germ free mice. The authors suggest that Tγδ cells may be a therapeutic target in acute lung injury dominated by neutrophilic inflammation. Walker et al. use a model of chronic inflammation in macaques treated with cART cells to suppress infection with SIV. In these macaques, although SIV infection is resolved, in the medium term there is a deterioration of the intestinal barrier and high levels of pro-inflammatory cytokines and markers of damage to the gastrointestinal barrier are detected. These markers are associated with a decrease in IL-17/IL-22-producing Tγδ cells.

### The epithelium as part of the innate immune system

Epithelial cells have developed a barrier function that prevents the entry of microorganisms and the translocation of potentially harmful antigens into the body. This barrier is based on the expression of adhesion molecules by epithlelial cells that form tight junctions between them. Lei et al. describe the importance of EpCAM-mediated adhesion of epithelial cells. These adhesion molecules control the expression of the polymeric immunoglobulin receptor (pIgR), resulting in a defect in transepithelial IgA transport in EpCAM KO mice. In addition, there is an increase in the expression of genes related to the inflammatory response responsible for the development of inflammatory bowel disease (IBD). On the other hand, the intestinal epithelium is adapted to an environment of hypoxia, which stimulates epithelial cells to produce TNF. Under these conditions the epithelium is more permeable, and the cells of innate immunity produce pro-inflammatory mediators. Kim et al. use a hypoxia-inducible factor (HIF) stabilizer in the model of DSS-induced colitis. It achieves the restoration of the epithelial barrier. The review by Kinashi and Hase discusses the role of the gut microbiota in the epithelial barrier. It describes how the weakening of this barrier contributes to the so-called leaky gut syndrome (LGS), which initiates an inflammatory response in the gut. A whole series of molecules produced by the commensal microbiota are critical in maintaining the epithelial barrier, so dysbiosis can be associated with intestinal inflammation and autoimmune diseases.

Homeostasis of the gut epithelium is maintained by an intestinal stem cell (ISC) compartment that resides at the base of intestinal crypts, giving rise to specialized epithelial cell lineages. Wisniewski et al. review the role of AhR ligands in ISC function and regulation. Tryptophan catabolites and SCFAs produced by the bacterial fermentation of dietary protein and soluble fiber serve as AhR ligands. Moreover, tissue stem cells have immunoregulatory properties and anti-inflammatory effects. Wu et al. describe the effect of conditioned medium of human amniotic epithelial cell cultures in experimental allergic conjunctivitis, mainly *via* IL-1ra and IL-10. Finally, mucosal barriers are also subjected to metabolic regulation. Qi et al. review the dual effect of endogenous ketogenesis in the microbiota, innate and adaptive immune systems, and the chemical and physical barriers of the mucosa.

### The adaptive immune system at mucosal surfaces

The mucosal membranes are colonized by a large number of microorganisms. Many of them live in symbiosis with the organism (commensal microbiota) but others are pathogenic. Host adaptation to the microbiota is an active process that involves interactions between immune cells and a balance between different types of lymphocytes. The adaptive immune system represents the most powerful defense against microorganisms and confers long-term memory. An effective defense against pathogens involves the effective mobilization of effector lymphocytes to the mucosal surfaces. The trafficking of effector T lymphocytes to mucosal surfaces is governed by the expression of homing receptors. The article by Manhas et al. studies the effect of rexinoids, which bind to nuclear receptors and promote the expression of integrins and chemokine receptors, favoring the migration of lymphocytes. They propose them as therapeutic agents in pathogenic infections or cancer on mucosal surfaces. Different classes of CD4+ lymphocytes, such as Th1, Th2, Th17, Th22, as well as regulatory T cells (Treg), B cells and antibody-producing cells, are part of the adaptive immune barrier of the mucosal surfaces. IgA is the component of the humoral response characteristic of these locations. The review by Keppler et al. describes how 80% of the cells that secrete IgA are found in the intestine, although they are also found elsewhere, especially the bone marrow. IgA represents an effective protection against enteric pathogens, but it is also effective in other locations, preventing sepsis and infiltration into other organs of pathogens from the mucosal surfaces. The review by Runge and Rosshart describes how different classes of microorganisms promote the appropriate immune response to each microorganism. The authors summarize the current knowledge on how microbes of all kingdoms and microbial niches, as well as some multicellular organisms, shape local and systemic immunity in health and disease. Each microorganism induces a particular type of response not only in the mucosal surfaces, but in the whole body. The article by Roy et al. describes that segmented filamentous bacteria induce not only Th17 cells, crucial in antimicrobial defense, but also IL-22-producing T cells that do not secrete IL-17 (bona fide Th22).

The balance between the different populations of T cells decides the balance between homeostasis and inflammation, not only in the mucosal surfaces but also throughout the organism. Imbalance in the proportion of T cell subsets is associated with inflammatory diseases, both in the digestive and respiratory systems, and is associated with autoimmune diseases such as Crohn’s disease or rheumatoid arthritis. Treg cells are key to this balance and are involved in immune tolerance. Fernandez-Perez et al. describe how T cell populations are altered in the absence of galectin 1, a lectin expressed in epithelial cells and different types of immune cells. In the DSS-induced gut inflammation model, the absence of galectin 1 induces increased inflammation, associated with an altered Th17/Th1 profile of effector CD4+ T cells, which is reduced after adoptive transfer of wild type Foxp3+CD4+ regulatory T cells. Ssemaganda et al. also show that the abundance of Treg in the genital tract is inversely associated with lymphocyte infiltration and inflammation of this location. Treg cells also play an important role in the repair processes after tissue injury. This is described by Tan et al. in the case of acute respiratory distress syndrome, in which the role of IL-33, an alarmin produced by damaged epithelial cells, in the expansion and activation of Treg and ILC2 is associated with lung repair.

The immune system of mucosal surfaces is subject to influences by the microbiome, epithelial cells, and nervous and endocrine systems. In this last aspect it should be noted that the gastrointestinal tract is highly innervated by the parasympathetic and sympathetic nervous systems. All these nerve terminals are in proximity to the lymphoid tissue at this location. Immune cells express receptors for nerve mediators, indicating integrated neuroimmune communication of particular importance in the gut. Vasoactive intestinal peptide (VIP) is a neuropeptide very abundant in this location and has important immunoregulatory properties. Leceta et al. collect previous knowledge and new data in a spontaneous model of rheumatoid arthritis and advance the hypothesis that VIP inhibits the plasticity of Th17 cells towards non-classical Th1 cells and enhances the function of follicular Treg cells. On the other hand, the article by Müller et al. study samples of patients affected by Hirschsprung disease, a neurodevelopmental congenital disorder in which there is a lack of ganglia of the intrinsic enteric nervous system. They show that the presence of cholinergic innervation attenuates the secretion of pro-inflammatory cytokines in intestinal epithelial cells. Webber et al. review the presence of hormone receptors in the lungs focusing on the effect that hormones have on the pulmonary immune response. One organ whose epithelial function is regulated by hormones is the uterus. The article by Han et al. describes the variations in the lymphoid population in the proliferative and secretory phases of the uterine epithelium cycle, also associated with changes in the microbiota at this location.

Other articles describe immune cells in other locations, such as the cornea (Li et al., Liu and Li) and the participation of the immune system in repair processes at serous surfaces (Zwicky et al.).

## Immunoregulatory role of the microbiota

Most of the microbiota is associated with the gastrointestinal tract but also colonizes other anatomical regions. Schubert et al. present a summary of the immuno-regulatory properties of the commensal microbiota, which synthesizes vitamins and regulatory products from the fermentation of dietary products, such as SCFAs, which stimulate the expansion and immunosuppressive properties of Treg lymphocytes, as well as other products that act on dentritic cells to decrease their immune-stimulating properties. Sabatini et al. describe how particular components of the microbiota, specifically Saccharomyces cerevisiae, promote the differentiation of a subtype of plasmacytoid dendritic cell that induce Th cells that produce IL-10. In this way, the authors describe how this yeast mediates the balance between pro- and anti-inflammatory responses. The microbiota is essential in the ontogeny, maturation, and modulation of immunity, both in the mucous membranes and systemically. An imbalance (dysbiosis) can lead to the exacerbation of inflammatory diseases and the development of autoimmune diseases.

In addition to the commensal microbiota, other species are pathogenic. The pathogenicity of these species begins with their adhesion to epithelial cells. Some species become pathogenic under certain conditions. Yang et al. in their article describe the case of one of these species, Helicobacter pylori, and how adhesion and colonization depend on the expression of Lewis antigens, which are induced by activation of the MAPK pathway. Conserved molecules of microorganisms are recognized by host-specific receptors (TLRs) in both epithelial cells and cells of the innate immune system, triggering immune responses. Pastille et al. study the involvement of TLR4 in the progression of inflammatory colitis and colorectal cancer and block the signaling pathway of this receptor with a specific inhibitory small molecule. Results show a significant reduction in the production of pro-inflammatory cytokines after inducing colitis *in vivo* with DSS or *in vitro* stimulation with LPS. In this way, tumorogenesis associated with inflammation is inhibited. Other resident species, known as pathobionts, have pathogenic potential, although they can establish a commensal relationship with the host promoted by factors derived from it. This is the case of Listeria monocytogenes reported by Cho et al. In this study it is described that the induction of specific CD8+ lymphocytes inhibit the expression of virulence factors in L monocytogenes.

Two articles are dedicated to the study of the evolution of the microbiota from birth and its involvement in the maturation of the immune system. Kalbermatter et al. provide a review of the impact of the microbiota from the gestation period to weaning. Environmental factors of early life, including the mode of birth, type of lactation, or use of antibiotics, modulate the maturation of the microbiota and the immune system in the first part of the development. Alterations of this dialogue between microbiota and immunological maturation of the newborn can have a long-term effect on susceptibility to diseases such as asthma or autoimmune diabetes. On the other hand, Schlosser-Brandenburg et al. in their study point out the importance of breastfeeding and physical closeness in the successful transfer of the maternal microbiota. Maternal isolation and formula lactation resulted in less bacterial diversity in the respiratory tract and less differentiation of Th1 activities in the lungs.

Finally, it should be noted that the importance of dysbiosis in the development of a whole series of diseases, especially gut inflammatory diseases, has led to the control of inflammation through the transplantation of fetal microbiota (FMT). Ponce-Alonso et al. address the issue of donor selection for FMT in ulcerative colitis (UC) remission. The selection of the donor was made after determining the degree of response of the immune cells of the recipient’s mucosa. Given the lack of results obtained in the evolution of UC of transplanted patients, they conclude that the selection of the donor based on this criterion is not the appropriate method to choose the donor for FMT.

## Author contributions

All authors co-edited equally this Research Topic, participating in the review of submissions. JL wrote this editorial article.

## Funding

This work was supported by grants from the European Research Council Starting Grant (ERCEA; 803087 to CSNK), the German Research Foundation (DFG; Project-ID 259373024 – CRC/TRR 167, B05 – CRC/TRR 241, FOR2599 project 5 - KL 2963/5-2, SPP1937 - KL 2963/2-1 and KL 2963/3-1 to CSNK).

## Conflict of interest

The authors declare that the research was conducted in the absence of any commercial or financial relationships that could be construed as a potential conflict of interest.

## Publisher’s note

All claims expressed in this article are solely those of the authors and do not necessarily represent those of their affiliated organizations, or those of the publisher, the editors and the reviewers. Any product that may be evaluated in this article, or claim that may be made by its manufacturer, is not guaranteed or endorsed by the publisher.

